# Combined Environment Simulator for Low-Dose-Rate Radiation and Partial Gravity of Moon and Mars

**DOI:** 10.3390/life10110274

**Published:** 2020-11-06

**Authors:** Akihisa Takahashi, Sakuya Yamanouchi, Kazuomi Takeuchi, Shogo Takahashi, Mutsumi Tashiro, Jun Hidema, Atsushi Higashitani, Takuya Adachi, Shenke Zhang, Fady Nagy Lotfy Guirguis, Yukari Yoshida, Aiko Nagamatsu, Megumi Hada, Kunihito Takeuchi, Tohru Takahashi, Yuji Sekitomi

**Affiliations:** 1 Gunma University Heavy Ion Medical Center, 3-39-22 Showa-machi, Maebashi, Gunma 371-8511, Japan; 3ra9di8o.y@gmail.com (S.Y.); tashiro@gunma-u.ac.jp (M.T.); skywatcher.tak@gmail.com (T.A.); m1920602@gunma-u.ac.jp (S.Z.); fadynagy.lotfy@yahoo.com (F.N.L.G.); yyukari@gunma-u.ac.jp (Y.Y.); 2 Matsuo Industries, Inc., 27-1, Ida, Kitasaki-machi, Obu, Aichi 474-0001, Japan; ka-takeuchi@kk-matsuo-ss.co.jp (K.T.); s-takahashi@kk-matsuo-ss.co.jp (S.T.); k-takeuchi@kk-matsuo-ss.co.jp (K.T.); t-takahashi@kk-matsuo-ss.co.jp (T.T.); y-sekitomi@kk-matsuo-ss.co.jp (Y.S.); 3 Division for the Establishment of Frontier Sciences of the Organization for Advanced Studies, Tohoku University, 2-1-1 Katahira, Aoba-ku, Sendai, Miyagi 980-8577, Japan; j-hidema@ige.tohoku.ac.jp; 4 Graduate School of Life Sciences, Tohoku University, 2-1-1 Katahira, Aoba-ku, Sendai, Miyagi 980-8577, Japan; atsushi.higashitani.e7@tohoku.ac.jp; 5 Japan Aerospace Exploration Agency, Tsukuba Space Center, 2-1-1 Sengen, Tsukuba, Ibaraki 305-8505, Japan; nagamatsu.aiko@jaxa.jp; 6 Radiation Institute for Science & Engineering, Prairie View A&M University, Prairie View, TX 77446, USA; mehada@pvamu.edu; 7 Material Solutions Center, Tohoku University, 2-1-1 Katahira, Aoba-ku, Sendai, Miyagi 980-8577, Japan

**Keywords:** combined effect, low-dose-rate radiation, partial gravity, simulator, Moon, Mars, neutron

## Abstract

Deep space exploration by humans has become more realistic, with planned returns to the Moon, travel to Mars, and beyond. Space radiation with a low dose rate would be a constant risk for space travelers. The combined effects of space radiation and partial gravity such as on the Moon and Mars are unknown. The difficulty for such research is that there are no good simulating systems on the ground to investigate these combined effects. To address this knowledge gap, we developed the Simulator of the environments on the Moon and Mars with Neutron irradiation and Gravity change (SwiNG) for in vitro experiments using disposable closed cell culture chambers. The device simulates partial gravity using a centrifuge in a three-dimensional clinostat. Six samples are exposed at once to neutrons at a low dose rate (1 mGy/day) using Californium-252 in the center of the centrifuge. The system is compact including two SwiNG devices in the incubator, one with and one without radiation source, with a cooling function. This simulator is highly convenient for ground-based biological experiments because of limited access to spaceflight experiments. SwiNG can contribute significantly to research on the combined effects of space radiation and partial gravity.

## 1. Introduction

NASA has planned to return to the Moon by 2024 with a mission named Artemis. The mission will start building the Lunar Gateway and aims to be a trial for deeper space exploration. It will be an important step for landing humans on Mars. It is necessary to develop a simulator of the environments on the Moon and Mars because the difference environment of space radiation and gravity condition in deep space as compared with Low Earth Orbit (LEO).

Space radiation environment is one of major risk factor in long-term exploration by humans. During LEO, the astronauts are protected from various space radiation by the Earth’s magnetic field. When they fly into the deep space, they need to pass through the trapped radiation belt composed with electrons and protons, and will be constantly exposed to solar particle events (SPEs) and galactic cosmic rays (GCRs), including high-energy heavy ions, without protection [1]. The longer the astronauts stay in deep space, the more the exposure to space radiation will be increased. No current spacecraft or spacesuit can shield astronauts from these energetic cosmic rays. Therefore, we need the ground-based experiments to simulate the space radiation environment. We can use the high-energy heavy-ion accelerators at specialized facilities such as the BEVALAC at the Lawrence Berkeley National Laboratory, the Heavy Ion Medical Accelerator in Chiba (HIMAC) at the National Institute of Radiological Sciences of the National Institutes for Quantum and Radiological Science and Technology (QST-NIRS), the GSI Helmholtz Center for Heavy Ion Research (GSI), and our Gunma University Heavy Ion Center (GHMC) [2] to expose biological samples. The majority of studies performed at these facilities were acute exposure of single radiation, fractioned doses, or combined sequential beams. In the NASA Space Radiation Laboratory (NSRL) at Brookhaven National Laboratory (BNL), SPE and GCR simulated beam can be used by rapid switching technology of ion species and ion energies [3]. Although we know that dose rates of space radiation are very low, it is challenging to perform exposure experiments with a low dose rate owing to the limited beam time allowed at such facilities. Recently, a concrete-shielded building on the campus of Colorado State University (CSU) was retrofitted with a panoramic Californium-252 (^252^Cf) source to allow low-dose-rate irradiation. The shielded vault can accommodate caging to simultaneously irradiate 900 mice and 60 rats for durations up to 400 days at a neutron dose rate of 1 mGy/day [4]. Long-term exposure to neutrons is a potential health hazard when astronauts encounter GCRs during their missions outside Earth’s magnetosphere. Despite that neutrons constitute a small proportion of GCRs, secondary neutrons ejected owing to interaction between GCRs and the shield elements are significant [5,6]. A major indicator is that the dose-averaged linear energy transfer (LET) of the low-dose-rate neutrons directly emitted from the ^252^Cf on Earth is close to that of the highly charged ions in space radiation. Furthermore, owing to the fundamental impact of primary and secondary scattered photons, high-energy neutrons emitted from ^252^Cf decomposition resemble the secondary ionizations of delta particle beams emitted from heavier high-energy charged particles [7]. Although they are a good substitute for the complex radiations that would be encountered in deep space, this ground experiment is in Earth’s gravity of 9.81 ms^−2^ (1*G*).

Partial gravity environments on the Moon and Mars are important to promote life science research, not only simulates microgravity (μ*G*) in spaceflight environments. Advanced ground-based systems for simulating gravity alterations have made it possible to study the response of living beings to altered gravity to prepare for experiments in space. Media that reduce vertical acceleration to the partial gravity of the Moon (0.165*G* ≒ 1/3*G*) and Mars (0.378*G* ≒ 3/8*G*) or other celestial body can achieve the μ*G* level or weightlessness [8]. Although a ballistic rocket, parabolic flight, or drop tower can produce a μ*G* environment for free fall on the Earth, the disadvantages of these approaches are the limited time of exposing to μ*G* and the additional hypergravity. To simulate μ*G*, researchers have used rotating devices such as the Rotating Wall Vessel bioreactor (RWV: Synthecone, Houston, TX, USA) and the Random Positioning Machine (RPM: Dutch Space, Netherlands). These are components of devices that revolve the sample in an uninterrupted manner. These machines can oppose and balance the direction of gravity and diminish its impact, inducing μ*G* [2]. Two main drawbacks to this concept are (i) revolution stops amid irradiation because the sample is susceptible to irradiation outside the incubator after or before rotation with an RWV [9,10,11,12,13], and (ii) the dose flatness in the irradiation area is non-uniform owing to irradiation from outside an RPM [14,15]. To deal with these limitations, we developed the System of Simultaneous irradiation in Simulated-μ*G* (SSS) using a three-dimensional (3D) clinostat [16,17]. The SSS relies on X-ray irradiation with a high-speed shutter [16], and on carbon-ion irradiation with radiological technologies like accelerator systems and respiratory gating systems [17].

**Figure 1:**
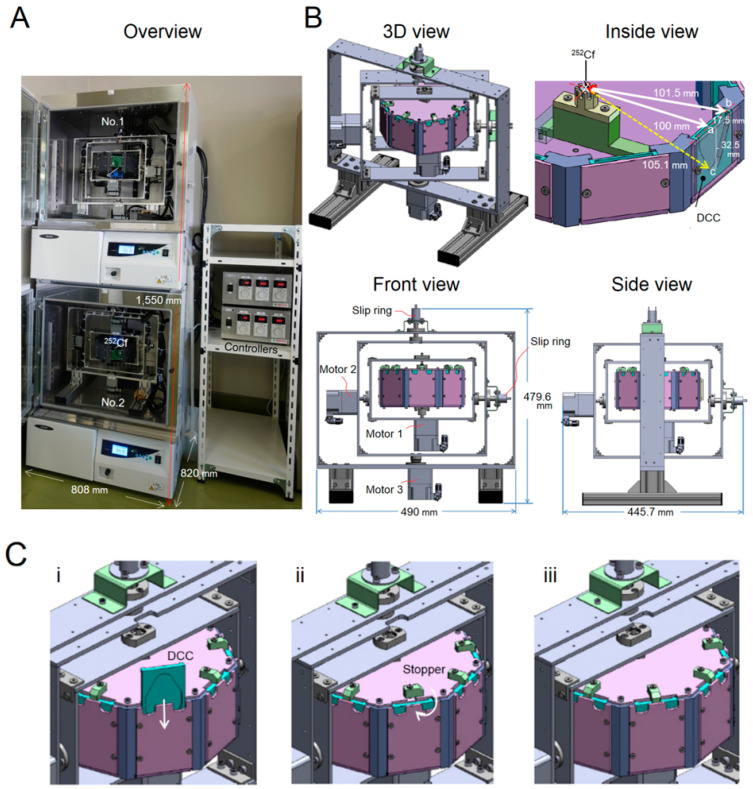
Schema of the Simulator of the environments on the Moon and Mars with Neutron irradiation and Gravity change (SwiNG). (**A**) Overview. (**B**) Outside view, and inside view of the rotor, where “x” is the radiation source, and “a” is the center, “b” is the short end, and “c” is the long end of the disposable closed cell culture chamber (DCC). (**C**) Setting a culture chamber in the rotor, which consists of (**i**) injecting the DCC and (**ii**) turning the stopper, after which (**iii**) the installation is complete.

More than 50 years ago, a relatively large centrifuge outfitted with clinostats was used for experiments simulating partial gravity [18]. Other researchers applied a simple angled or inclined clinostat to generate partial gravity [19,20], and systems like centrifuge-clinostats were constructed [21,22]. Recently, researchers have developed devices using the RPM to simulate partial gravity [8]. As a next step, we need to investigate the effects of partial gravity on the biological response to radiation for manned missions to the Moon and Mars and compare them with those of both simulated μ*G* and Earth gravity (1*G*). In this study, we develop the new Simulator of the environments on the Moon and Mars with Neutron irradiation and Gravity change (SwiNG) (Figure 1).

## 2. Materials and Methods

### 2.1. System Descriptions

The simulator is comprised of five subsystems: (1) a 3D clinostat, (2) a centrifuge, (3) a radiation source, (4) a sample chamber [23], and (5) an incubator. Its specifications are summarized in Table 1. The compact system (W 808 mm × D 820 mm × H 1550 mm) has two SwiNG devices, one with and one without a ^252^Cf source in the incubators with a cooling function.

### 2.2. Neutron Source

We calculated the amount of ^252^Cf required to provide a space-relevant dose rate of 1 mGy/day according to Borak et al. [4]. The shielding guide DP-1246 quotes the fission neutron yield as 2.4 × 10^12^ s^−1^ g^−1^ with a corresponding photon yield of 1.3 × 10^13^ s^−1^ g^−1^ [24]. The configuration of the facility was based on a panoramic irradiator encircled by a disposable closed cell culture chamber (DCC) at a radius of 100 mm from the radiation source. DP-1246 was used to estimate the initial quantity of ^252^Cf (neutron) required to provide a space-relevant dose rate of 1 mGy/day. Using the neutron yield for ^252^Cf, the fluence rate at 100 mm (Φn) is

Φn = 2.4 × 10^12^ s^−1^ g^−1^ ÷ (4π × 100 mm × 100 mm) = 19.1 × 10^6^ mm^−2^ s^−1^ g^−1^ (1)

DP-1246 lists the dose rate conversion factor (*K*n) for soft tissue in a phantom approximating a man as

*K*n = 1.43 × 10^−2^ mGy h^−1^ mm^2^ s (2)

The neutron dose rate at 100 mm (*D*n) would be

*D*n = Φn × *K*n = 19.1 × 10^6^ mm^−2^ s^−1^ g^−1^ × 1.43 × 10^−2^ mGy h^−1^ mm^2^ s = 27.3 × 10^4^ mGy g^−1^ h^−1^ (3)

The desired neutron dose rate based on an exposure time of 24 h/day is

*D*n = 1 mGy ÷ 24 h = 4.2 × 10^−2^ mGy h^−1^ (4)

Thus, the required amount of ^252^Cf is

^252^Cf = 4.2× 10^−2^ mGy h^−1^ ÷ (27.3 × 10^4^ mGy g^−1^ h^−1^) = 15.4 × 10^−8^ g (5)

The required activity of ^252^Cf is

^252^Cf Bq = (ln2 ÷ T s) × (W g ÷ M g mol^−1^) × N_A_ mol^−1^         = (0.693 ÷ (8.3 × 10^7^ s)) × (15.4 × 10^−8^ g ÷ 252 g mol^−1^) × 6.0 × 10^23^ mol^−1^ = 3.1 × 10^6^ s^−1^ = 3.1 × 10^6^ Bq = 3.1 MBq       (6)

where, T is the half-life (s), W is the weight (g), M is the molar mass (g mol^−1^), and N_A_ is Avogadro’s constant (mol^−1^). 

The unregulated sealed source containing 3.7 MBq ^252^Cf was delivered on 17 March 2020 from Japan Radioisotope Association (Tokyo, Japan). The ^252^Cf was used in accordance with the Recommendations for Regulation on Prevention of Ionizing Radiation Hazards, compiled by the Prevention of Ionizing Radiation Hazards Committee of Gunma University, Showa Campus.

### 2.3. Calculation of Dose Distribution

To determine the quantity of ^252^Cf necessary to achieve 1 mGy/day, a series of Monte Carlo simulations were conducted using the Particle and Heavy Ion Transport code System (PHITS) version 3.17 [25,26].

### 2.4. Measurement of Dose Rate

**Figure 2:**
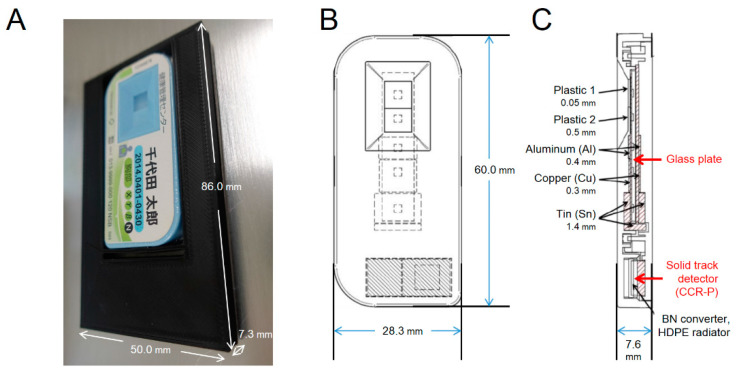
Glass badge dosimeter unit. (**A**) Overview of dosimeter in a special holder. (**B**) Front view of dosimeter’s internal structure. (**C**) Side view of dosimeter’s internal structure (technical data from Chiyoda Technol Co., Tokyo, Japan).

The dosimeter (radiophoto-luminescence EN, Chiyoda Technol Co., Tokyo, Japan) was inserted into a special holder (Adachi Factory, Gunma, Japan) (Figure 2) and attached to an octagonal rotor as same as sample position with and without ^252^Cf for 24 h. The alpha rays generated by the interaction of neutrons with special filters consisted of high density polyethylene and boron nitride, and anti-jump protons generated by the reaction of neutrons with hydrogen atoms are used in the Wide-range Neutron Pit System (Chiyoda Technol Co., Tokyo, Japan). The neutron dose rate was measured indirectly by alpha rays and protons, and analyzed by Chiyoda Technol Co. using ISO8529. The data presented here are from three individual experiments.

### 2.5. Measurement of Gravity Using Accelerometer

A 3D accelerometer (ADXL335, Analog Devices, Norwood, MA, USA) was used to measure the gravitational accelerations at the sample position along the X, Y, and Z directions in the octagonal rotor. The accuracy of this sensor is ± 0.0069*G*. The gravitational accelerations were analyzed using original software (Matsuo Industries, Inc., Aichi, Japan).

### 2.6. Measurement of Temperature

A K-type thermometer (K-H0.1X1P, Ninomiya Electric Wire Co., Kanagawa, Japan) and a data logger (NR-600, KEYENCE Co., Osaka, Japan) were used to measure and record the temperatures of the centrifuge motor and the incubator including the device.

### 2.7. Statistical Analysis

Statistical analysis was performed using the Student *t*-test. *p*-values of less than 0.05 were considered statistically significant in comparing samples.

## 3. Results

### 3.1. Simulation of Flatness and Symmetry in the Irradiation Fields

**Figure 3:**
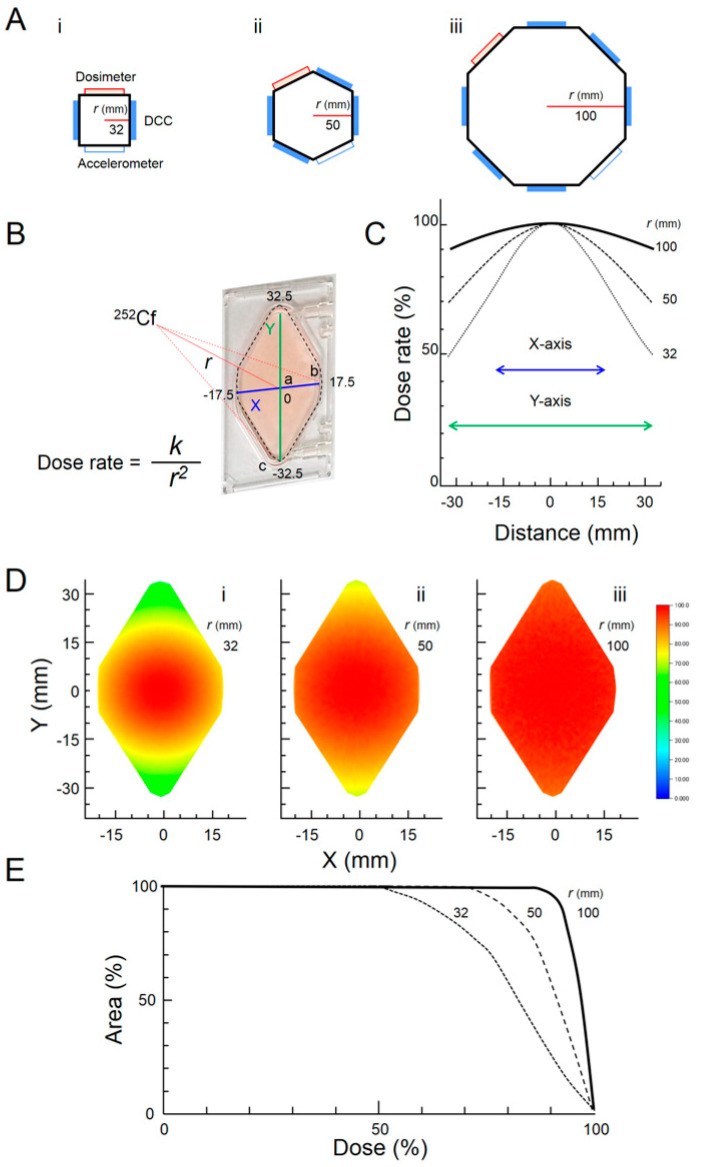
Theoretical radiation dose against rotor size. (**A**) Rotor: (**i**) square, (**ii**) hexagonal, and (**iii**) octagonal. (**B**) DCC. Dashed lines indicate actual positions of cells in a culture chamber, where “a” is the center, “b” is the short end, and “c” is the long end of the DCC. (**C**) Relative dose against distance from the intersection of the X and Y axes; the relative dose is 100% at the intersection of X and Y. (**D**) Dose distribution simulated with PHITS. (**E**) Dose area histogram.

The dose distribution of the samples was uniform along the X and Y axes (Figure 3). The assumed radius of the center was 32, 50, and 100 mm in the square, hexagonal, and octagonal rotor (Figure 3A). The square, hexagonal, and octagonal rotors took two, four, and six DCCs, respectively, in addition to a dosimeter and an accelerometer. The dose rate is inversely proportional to the square of the distance from the radiation source (Figure 3B). When it is away from the center in the X and Y directions, the relative dose rate of the DCC was shown in Figure 3C. The radiation dose distribution was simulated with PHITS for each rotor (Figure 3D). These results suggest that the octagonal rotor is the ideal choice for uniform dose distribution of radiation. The radiation covers a greater total DCC area with the octagonal rotor than with the hexagonal rotor, which in turn achieves more total area radiation coverage than the square rotor (Figure 3E). Based on the data, we have decided to adopt the octagonal rotor in SwiNG.

The estimated data in Table 2 confirm that the dose uniformly covers the DCC area (Figure 3C). With point a in Figure 3B being the intersection of the X and Y axes and receiving 100% of the dose, the difference in dose delivery between point b (the short end) and point a (the center) is less than 3%, and the difference in dose delivery between point c (the most distant long-end point) and point a is less than 10%, confirming uniform dose delivery (Table 2).

### 3.2. Measurement of Dose Rate

The measured dose rates were comparable with the theoretical dose rate about 1 mGy/day at the center of the sample in the octagonal device with ^252^Cf (No. 2) within the nominal range (Table 2 and Table 3). The dose rate of γ-ray by the disintegration of ^252^Cf was 0.5 ± 0.1 mGy/day at the sample position.

Since the control samples in the device without ^252^Cf (No. 1) are ca 800–1000 mm away from the ^252^Cf in No. 2 incubator, the dose rate of control sample is assumed at least 1/64–1/100 that of the irradiation sample in the device with ^252^Cf (No. 2). In fact, the dose rate of control group in the device without ^252^Cf (No. 1) was below the detection limit. The data of environmental radiation monitoring around SwiNG was shown in Table 4.

### 3.3. Simulation of Flatness and Symmetry in the Gravity Fields

For the octagonal rotor, the assumed distances from the center axis of the centrifuge were 100 mm and 100–101 mm along the Y and X axes of the sample. The theoretical relative centrifugal force (RCF) distributions of the samples were equal along the Y axis and almost equal along the X axis.

### 3.4. Measurement of Gravity

**Figure 4:**
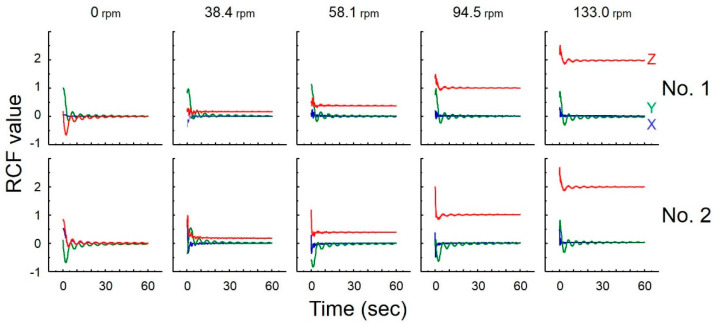
Measured integrated RCF for different rotary speeds of motor 1 during 1-min rotation. The rotary speeds of motors 2 and 3 were 13.0 and 11.0 rpm, respectively.

The simulator could reproduce from μ*G* up to 2*G*. At each rotation speed, the theoretical and measured RCFs of SwiNG nos. 1 and 2 were almost the same (Table 5 and Figure 4). After about 20 s, the measured integrated RCF was very stable (Figure 4).

### 3.5. Measurement of Temperature

Although the temperature of the centrifuge motor at 133.0 rpm was the maximum of 38 °C, the temperature in the incubator including the device was 37 ± 0.2 °C when set to 37 °C (data not shown). As the sample holder of the rotor was made of plastic (polybutylene terephthalate—30% glass fiber, PBT-GF30), heat from the motor did not conduct to the samples.

## 4. Discussion

The new compact simulator, SwiNG, was developed to expose samples to low-dose-rate neutron radiation (1 mGy/day) from ^252^Cf under partial gravity using a centrifuge (r = 100 mm) in a 3D clinostat (Figure 1). Using a centrifuge (r = 100 mm), the PHITS simulation confirmed our system’s ability to provide uniform dose distribution (Figure 3Diii). In our new system, radiation source is located at the center of the chamber in the rotor, and surface of the samples are constantly facing to the radiation source. Although we measured the dose rate at center of sample position only at 1*G* (Table 3), it is unlikely that the dose will change under any gravity for each of the centrifuge cases. The reasons were that the distance between the neutron source and the sample is constant at 100 mm. Further, motors 2 and 3 are constantly rotating under each gravitational condition, so that the effects of scattered radiation in the incubator is uniform on the samples. Interesting results have been reported with mice exposed to low-dose neutrons using ^252^Cf at CSU [28,29,30]. New discoveries can be expected with our new simulator system.

We confirmed that the system can instantaneously change the gravity environment (Figure 4) in addition to providing low-dose radiation exposure. The rotation speed of the centrifuge can be changed in accordance with the user’s purpose and choice of samples to simulate partial gravity. Moreover, the simulator can reproduce μ*G*, the Earth control of 1*G*, and hypergravity condition (up to 2*G*) of launch and landing to Earth. Another advantage of our new system is that six samples can be exposed to radiation at the same time.

We are planning to use DCCs for in vitro biological experiments such as DNA damage, chromosomal aberrations, gene expression changes, and so on using cultured cells. The chamber can be completely filled with a medium (without bubbles) to eliminate shear stress on the cells or minimize it as much as possible [31]. Shear stress is reported to be almost negligible in the absence of air bubbles in a slowly rotating medium [32]. We have already validated DCCs using human fibroblasts and lymphoblasts cultured with a CO_2_-independent medium (Thermo Fischer Scientific, Waltham, MA, USA) and found no problems with cell growth under atmospheric conditions [17,33,34]. This DCC chamber can exchange the gas thorough the special gas permeable membrane [23]. Therefore, the oxygen available inside the DCC is available from the atmosphere and is sufficient for cell metabolism, and CO_2_ will be supplied from buffered medium. Furthermore, the temperature of the sample can be easily controlled and maintained under suitable conditions in the incubators with a cooling function. Besides human cell cultures, the simulator can be used for microorganisms such as bacteria, yeast, *Pyrocystis noctiluca* or *Dictyostelium discoideum* which has been used in past ground-based experiments and/or space experiments [35,36,37,38,39,40].

To confirm experimental results obtained from in vitro experiments with our simulating system, it will be important to perform in vivo experiments with partial gravity such as quadrupedal unloading [41,42,43,44] on the ground. The effects should be tested either in a proper centrifuge experiment on the ISS, such as the Cell Biology Equipment Facility (CBEF) [45] and mouse habitat unit (MHU) cage [46], or on the actual surfaces of the Moon and Mars.

## 5. Conclusions

We developed the SwiNG for simulator of the environments on the Moon and Mars with low-dose-rate (1 mGy/day) irradiation and partial gravity, and evaluated the physical performance of the hardware using theoretical and measured values of neutron dose rate and averaged gravity at the sample position in octagonal rotator. The simulator is highly convenient for space biology research because of the limited availability of space experiments. The basic data from ground experiments using our system are expected to help develop a new biological parameter for evaluating human health risks in space radiation involving radiological protection.

## Figures and Tables

**Table 1:** Specifications of the Simulator of the environments on the Moon and Mars with Neutron irradiation and Gravity change (SwiNG).

Subsystem	Specifications
3D clinostat	External size: W 490 mm × D 445.7 mm × H 479.6 mm
	Rotational velocity: 66°/s and 78°/s
	Manufacturer: Matsuo Industries, Inc. (Aichi, Japan)
Centrifuge	Rotor size: W 216 mm × D 216 mm × H 90 mm (octagonal type)
	Speed control range: 27–133 rpm
	Manufacturer: Matsuo Industries, Inc.
Radiation source	^252^Cf (N-252CE, Japan Radioisotope Association, Tokyo, Japan)
	External size: Diameter 9.4 mm × L 36.3 mm
	Dose-equivalent average energy: 2.3 MeV
	Dose-averaged LET: 68 keV/μm
	Half-life: 2.645 years
Sample chamber	Disposable closed cell culture chamber (DCC, Chiyoda Co., Kanagawa, Japan)
	External size: W 86.0 mm × D 50.0 mm × H 7.3 mm
	Cultivation area: 15.5 cm^2^ (W 65 mm × D 35 mm)
	Liquid depth: 3 mm
	Material: polystyrene (bottom thickness: 1 mm; top thickness: 50 µm)
	Six DCCs can be placed in the rotor
Incubator	Low-temperature incubator without control of CO_2_ concentrations: LTE-510 (Tokyo Rikakikai Co., Tokyo, Japan)
	Internal size: W 600 mm × D 500 mm × H 500 mm
	Temperature control range/accuracy: −10–60 °C/±0.2 °C

**Table 2:** Theoretical neutron dose rate of 3.7 MBq ^252^Cf.

Position of DCC * in Octagonal Rotor	Distance from ^252^Cf	Theoretical Dose Rate ^†^	Relative Value
a (center)	100.0 mm	1.23 ± 0.18 mGy/day	100.0
b (short end)	101.5 mm	1.19 ± 0.18 mGy/day	97.1
c (long end)	105.1 mm	1.11 ± 0.17 mGy/day	90.5

* See Figure 1B and Figure 2C. ^†^ Nominal value ± 15%.

**Table 3:** Using glass badge, measured radiation dose equivalent at the control sample and the irradiation sample position of SwiNG for 24 h.

	Control Samples	Irradiation Samples
Incubator of SwiNG	No. 1	No. 2
Neutron *	ND	10.8 ± 0.0 mSv
Total dose rate ^†^	ND	1.08 ± 0.00 mGy/day

Measurement date, 18–21 March, 2020. * 1 cm dose equivalent. ND, not detected. ^†^ The dose rate was determined from the dose equivalent. The radiation weighting factor of neutron (^252^Cf, 2.1 MeV) was calculated as 10 [27].

**Table 4:** The environmental radiation monitoring around SwiNG.

Position	γ-ray	Neutron	Total
The surface of SwiNG	1.5 µSv/h	18.4 µSv/h	19.9 µSv/h
1 m distance from ^252^Cf	0.3 µSv/h	6.7 µSv/h	7.0 µSv/h
2 m distance from ^252^Cf	0.2 µSv/h	2.5 µSv/h	2.7 µSv/h

**Table 5:** Simulated relation between centrifuge rotation and gravity at the sample position of SwiNG.

Rotary Speed of Motor 1 (Centrifuge) *	Theoretical RCF ^†^ (*r* = 100–101 mm)	Measured RCF ^‡^		Simulation
		No. 1	No. 2	
0.0 rpm	~µ*G*	0.01 ± 0.00*G*	0.01 ± 0.00*G*	interplanetary space
38.4 rpm	0.165–0.167*G*	0.17 ± 0.00*G*	0.17 ± 0.00*G*	on the Moon
58.1 rpm	0.377–0.381*G*	0.38 ± 0.00*G*	0.39 ± 0.00*G*	on Mars
94.5 rpm	0.998–1.008*G*	1.01 ± 0.00*G*	1.01 ± 0.00*G*	on the Earth
133.0 rpm	1.978–1.997*G*	1.98 ± 0.01*G*	2.00 ± 0.00*G*	hypergravity

* The rotary speeds of motors 2 and 3 were 13.0 and 11.0 rpm, respectively. ^†^ RCF (Relative centrifugal force) = 1.118 × r [mm] × N^2^ [rpm] × 10^−6^. ^‡^ The presented results are the mean and SD of three independent experiments.
